# The impact of mutational clonality in predicting the response to immune checkpoint inhibitors in advanced urothelial cancer

**DOI:** 10.1038/s41598-023-42495-2

**Published:** 2023-09-15

**Authors:** Lilian Marie Boll, Júlia Perera-Bel, Alejo Rodriguez-Vida, Oriol Arpí, Ana Rovira, Núria Juanpere, Sergio Vázquez Montes de Oca, Silvia Hernández-Llodrà, Josep Lloreta, M. Mar Albà, Joaquim Bellmunt

**Affiliations:** 1 Hospital del Mar Research Institute, Barcelona, Spain; 2 Medical Oncology Department, Hospital del Mar, Barcelona, Spain; 3 Centro de Investigación Biomédica en Red de Oncología (CIBERONC-ISCIII), Barcelona, Spain; 4 Department of Medicine and Life Science, Universitat Pompeu Fabra (UPF), Barcelona, Spain; 5 Catalan Institute for Research and Advanced Studies (ICREA), Barcelona, Spain; 6 Dana Farber Cancer Institute, Harvard Medical School, Boston, MA USA

**Keywords:** Cancer genomics, Urological cancer, Cancer immunotherapy, Immunotherapy

## Abstract

Immune checkpoint inhibitors (ICI) have revolutionized cancer treatment and can result in complete remissions even at advanced stages of the disease. However, only a small fraction of patients respond to the treatment. To better understand which factors drive clinical benefit, we have generated whole exome and RNA sequencing data from 27 advanced urothelial carcinoma patients treated with anti-PD-(L)1 monoclonal antibodies. We assessed the influence on the response of non-synonymous mutations (tumor mutational burden or TMB), clonal and subclonal mutations, neoantigen load and various gene expression markers. We found that although TMB is significantly associated with response, this effect can be mostly explained by clonal mutations, present in all cancer cells. This trend was validated in an additional cohort. Additionally, we found that responders with few clonal mutations had abnormally high levels of T and B cell immune markers, suggesting that a high immune cell infiltration signature could be a better predictive biomarker for this subset of patients. Our results support the idea that highly clonal cancers are more likely to respond to ICI and suggest that non-additive effects of different signatures should be considered for predictive models.

## Introduction

Cancer is among the greatest burdens of disease in high- and middle-income countries, with urothelial cancer being the sixth most common cancer type in men globally^1^. Genetic aberrations play an important role in the development of urothelial cancer. Somatic variants resulting in mutated proteins can be processed and presented on the tumor cell’s surface as small peptides called neoantigens. Neoantigens are tumor-specific, and thus, have a selective potential for T cell recognition^2^. One way tumors cells evict immune escape is by overexpressing checkpoint inhibitors. When binding to the ligand on the T cell surface, an immune response is suppressed by downregulating the production of cytokines, effector functions and T cell proliferation^3^. In urothelial carcinoma, high expression of the checkpoint molecule programmed death-ligand1 (PD-L1) has been associated with advanced cancer stages and poor survival^4^.

Immune checkpoint inhibitors (ICIs) have been a major advance in immunotherapy in the past decade. Monoclonal antibodies block immune checkpoints to prevent tumor cells from escaping T cell recognition. To date, several PD-1/PD-L1 inhibitors have been approved by the FDA and EMA, for patients with advanced urothelial cancer. However, less than one-third of urothelial cancer patients respond to ICI treatment^5–8^. Accurate methods to predict which patients are going to respond to the treatment are currently missing and are urgently needed in the clinics.

Based on the assumption that a higher tumor mutational burden (TMB) translates to an increased neoantigen load, TMB is nowadays one of the most relevant biomarkers currently being studied for clinical use in urothelial cancer^7,9,10^. However, findings are inconsistent across studies, and the isolated use of TMB has not been shown to accurately differentiate between responders and non-responders^8,11,12^. One possible way to improve the accuracy of the predictions is to consider neoantigen quality in addition to quantity^13^. Several computational prediction programs exist to estimate characteristics of neoantigens such as the ability to form a stable complex with the major histocompatibility complex (MHC) receptor or the probability of immune cell recognition. Yet, experimental studies showed that many of these computationally predicted epitopes are not presented on the cell surface^14^, limiting the applicability of these approaches.

Previous efforts to investigate the impact of different biomarkers in the response to ICI have mostly centered on melanoma and lung cancer—the cancers with the largest number of mutations—or in the meta-analysis of different types of cancer. These studies have suggested that the clonality of the mutations might be especially relevant. For example, McGranahan et al. showed that sensitivity to PD-1 and CTLA-4 blockade in patients with advanced non-small cell lung cancer and melanoma was enhanced in tumors enriched for clonal neoantigens^15^. These findings are consistent with Wolf et al*.* who report that clonal neoantigens relate to immune infiltration and clinical outcome in melanoma^16^. Another study including patients with urothelial cancer treated with ICIs targeting PD-1/PD-L1 and CTLA-4 found that clonal mutations were significantly higher in complete responders compared to non-responders or partial responders^17^. Finally, Litchfield et al.^18^, who performed a meta-analysis of biomarkers from different cancer types, found that clonal TMB was the strongest predictor of CPI response followed by total TMB and CXCL9 expression. Subclonal TMB, somatic copy alteration burden, and histocompatibility leukocyte antigen (HLA) evolutionary divergence failed to attain pan-cancer significance.

In order to gain knowledge on what might drive the response to monoclonal antibodies blocking the programmed cell death-1 (PD-1) or its ligand (PD-L1) in advanced urothelial cancer, and how this compares to other kinds of cancers, we performed an in-depth study of whole exome and RNA sequencing data from a cohort composed of 27 patients treated with ICIs targeting PD-1/PD-L1 from Hospital del Mar in Barcelona (Spain). As an independent dataset, we also analyzed somatic variation in urothelial cancers from a 25 patients cohort treated with the PD-L1 inhibitor atezolizumab at Memorial Sloan Kettering Cancer Center^8^. The results provide additional novel insights into the genomic correlates associated with immunotherapy response and suggest ways in which predictive models could be improved.

## Results

### Genomic analysis and cohort description

**Figure 1:**
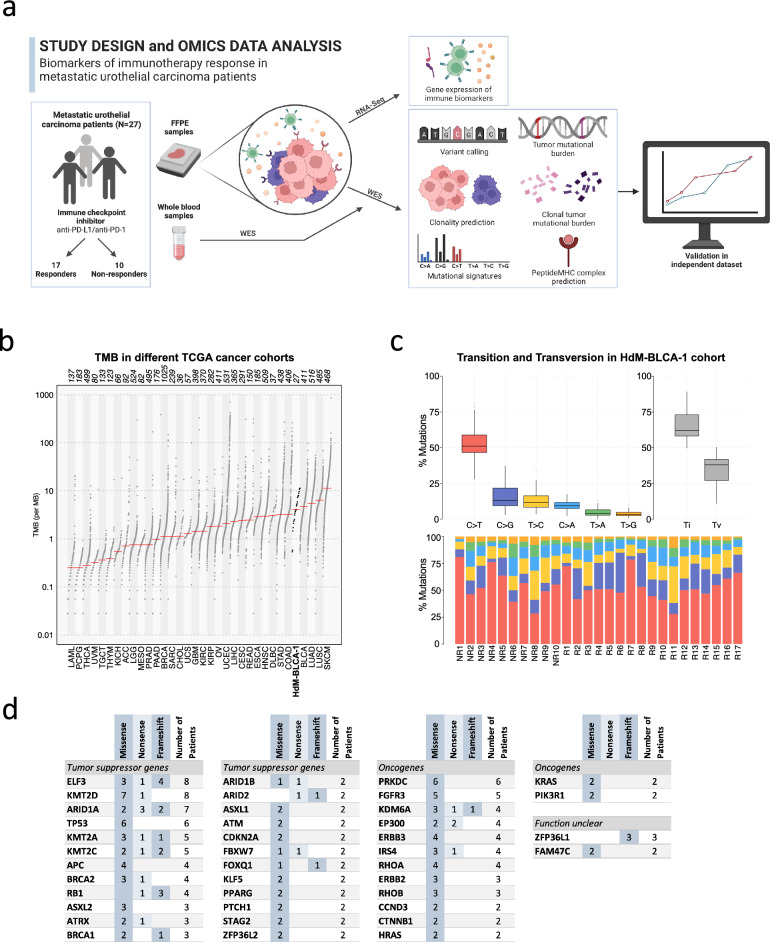
Analysis of omics data from the metastatic urothelial cancer cohort at Hospital del Mar. (**a**) Study design. We performed whole exome sequencing (WES) and RNA sequencing (RNA-Seq) from a cohort of 27 patients. The cohort included 17 responders (partial or complete response) and 10 non-responders (progressive disease). We measured the differences in the distribution of several biomarkers, including clonal and subclonal TMB, mutational signatures, number of predicted mutation-induced neoantigens and gene expression signatures in responders and non-responders. Created with BioRender.com. (**b**) Tumor mutational burden (TMB) compared to different TCGA cancer cohorts. The number of non-synonymous mutations in the cohort in our cohort (HdM-BLCA-1) was very similar to the bladder cancer TCGA cohort and in accordance with this being a highly mutated cancer. The names below the plot represent the TCGA cohort abbreviation, the numbers above represent the cohort sizes. (**c**) Frequencies of different pairwise nucleotide substitutions. C->T mutations were the most frequent ones, followed by C->G. *TI* transition, *Tv* transversion. (**d**) Cancer-related frequently mutated genes. Missense, frameshift and nonsense mutations are shown. Genes have been classified into ‘Oncogenes’, ‘Tumor suppressor genes’ and ‘Function unclear’ based on previous knowledge. *NR* no responders, *R* responders.

We generated whole-exome sequencing (WES) and RNA sequencing (RNA-Seq) data from tumors and blood samples from 27 advanced urothelial cancer patients treated with anti-PD-1/PD-L1 ICIs at Hospital del Mar (Fig. 1a). WES data was obtained from the tumors before treatment as well as from blood samples, allowing reliable identification of cancer-specific mutations. The type of response to treatment was defined using the Response Evaluation Criteria In Solid Tumors (RECIST) criteria 1.1. Among the 27 patients whose tumors were sequenced, 17 were responders to the ICI treatment (12 partial, 5 complete), and 10 were non-responders. Complete response is described as the complete disappearance of the tumor tissue, partial response a decrease of the target lesion by at least 30%. Additionally, we obtained high-quality RNA-Seq data from tumor samples in 20 patients of which 13 were responders to the ICI treatment (8 partial, 5 complete responses) and 7 were non-responders (progressive disease). The mutational data was used to investigate the effect of the number and type of mutations in response to treatment, as well as to predict putative neoantigens on a patient basis. The gene expression data was used to investigate the impact of different immune-based markers in ICI response.

### Estimation of tumor mutational burden and identification of recurrently mutated genes

Overall, we identified 6989 cancer-specific non-synonymous mutations. Most mutations were missense, 6192 (88.6%), but we also detected 490 nonsense (premature stop codons) and 130 frameshift mutations. We calculated the TMB for each patient as the sum of non-synonymous mutations. The average TMB of the dataset was 5.18 mutations per Mb, comparable to that observed in the cancer genome atlas (TCGA) bladder cancer cohort (Fig. 1b). Among pairwise nucleotide substitutions, C->T mutation was the most common (Fig. 1c), with a median frequency of around 0.5, very similar to that observed in the TCGA bladder cancer dataset (Figure S1) (18). The second most common mutation was C->G. We also specifically searched for the APOBEC signature, which has been previously found to be frequent in urothelial cancer^19,20^. APOBEC cytidine deaminases have the ability to introduce mutations in chromosomal and mitochondrial DNA potentially driving oncogenesis^21^. In the APOBEC signature C mutates to G or T in the context of TCW motifs (where W is A/G). Using the maftools package^22^, we found that this signature was significant in 16 out of 27 sequenced tumors.

We identified previously described driver and tumor suppression genes that were mutated in more than one patient in our cohort. These included genes in pathways related to TP53/cell cycle (*TP53, RBI, ATM*), histone modification (*KDM6A, EP300, KMT2A/C/D*), DNA damage (*BRCA1/2*) and chromatin remodeling (*ELF3, ARID1A, ARID1B, ARID*2)^19,23,24^ (Fig. 1d). The two most mutated genes were *ELF3* and *KMT2D*, with alterations in 8 patients. *ELF3* is an important transcriptional regulator for the differentiation of the urothelium with a high mutation frequency in bladder cancer^25^, and histone-lysine *N*-methyltransferase genes (*KMT2A/C/D*) are among the most mutated in different cancer types^26,27^. The majority of changes in *ELF3* were frameshift and nonsense mutations, similar to the findings of Nordentoft et al.^24^. In contrast, in *KMT2D*, 7 out of 8 mutations were missense.

### Tumor mutational burden is significantly associated with the response to ICI

**Figure 2:**
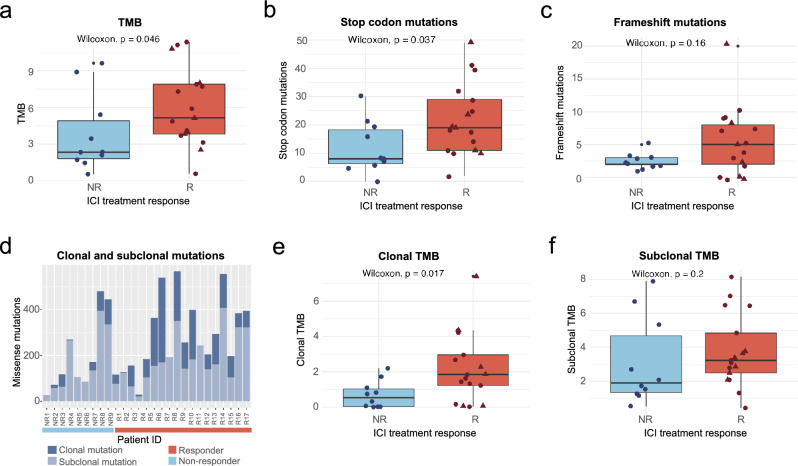
TMB and clonal TMB are significantly associated with the response to ICI. (**a**) Relationship between TMB and response to ICI treatment. TMB values were significantly higher in responders than in non-responders (Wilcoxon test, p value = 0.046). (**b**) Relationship between stop mutations and response to ICI treatment. Nonsense mutations were significantly higher in responders than in non-responder (Wilcoxon test, p value = 0.037). (**c**) Relationship between frameshift mutations and response to ICI treatment. Frameshift mutations showed a trend of being more abundant in responders than non-responders but the difference was not statistically significant (p = 0.3). (**d**) Number of mutations per patient divided in clonal and subclonal. The proportion of clonal mutations is significantly higher in responders (Fisher’s exact test p value = 0.007). (**e**) Relationship between clonal TMB and response to ICI treatment. Clonal TMB values were significantly higher in responders than in non-responders (Wilcoxon text, p value = 0.017). (**f**) Relationship between subclonal TMB and response to ICI treatment. Responders tend to have higher values but the difference between responders and non-responders is not statistically significant. *NR* no responders, *R* responders, triangle shape represents complete responders among the group of responders.

Next, we investigated the relationship between different types of mutations and the response to treatment. We found that the number of non-synonymous mutations or TMB was significantly higher in responders than in non-responders (Fig. 2a, Wilcoxon test p value = 0.046). This is in agreement with previous observations for different cancer types^17,18,28–30^. Additionally, we found that the number of nonsense mutations was also higher in responders than in non-responders (Fig. 2b). Responders also tended to have more frameshift mutations than non-responders, although in this case, the difference was not statistically significant (Fig. 2c).

### Clonal TMB better separates responders from non-responders than total TMB

Some mutations in the tumor are present in all cells (clonal), whereas others are only present in a subset of the cells (subclonal). We used the cancer cell fraction (CCF) statistic, in which the initial allele frequencies are corrected by tumor purity and copy number, to estimate the number of clonal mutations in each sample. Mutations with a CCF > 0.9 were considered clonal. This accounted for 2243 mutations, 1963 (87.5%) of which were missense mutations (Additional file 2). We observed that clonal mutations were overrepresented among recurrently mutated genes (see previous section): 73 out of 137 mutations in these genes were clonal. This is significantly higher than the expected number given that 28% of the mutations were clonal (Chi-square test, p < 0.0001).

We found multiple lines of evidence that clonal mutations had a disproportionately higher impact in the response to the treatment when compared to other types of mutations. First, the samples of responders were strongly enriched in clonal mutations when compared to those of non-responders; the average percentage of clonal mutations in responders was twice the percentage in non-responders (34.32% versus 17.34%, Fisher’s exact test p value = 0.007) (Fig. 2d). Second, when computing clonal TMB instead of total TMB, the differences between responders and non-responders clearly increased; for clonal mutations, the difference between the median values of the two groups showed an × 1.7 increase, and there was less overlap between the two distributions (Fig. 2e versus 2a). In contrast, there were no statistically significant differences in subclonal TMB between responders and non-responders (Fig. 2f, Wilcoxon test p value = 0.24). Third, we found that, for clonal mutations, there was an enrichment in APOBEC induced mutations in responders compared to non-responders (p value = 0.03, Figure S2); this effect was not observed when considering all non-synonymous mutations (p value = 0.44).

We checked if the above observations could be driven by the subset of complete responders. However, we did not observe a statistically significant difference in total, clonal or subclonal TMB when comparing complete responders and partial responders (Figure S3), indicating that this is not the case.

**Figure 3:**
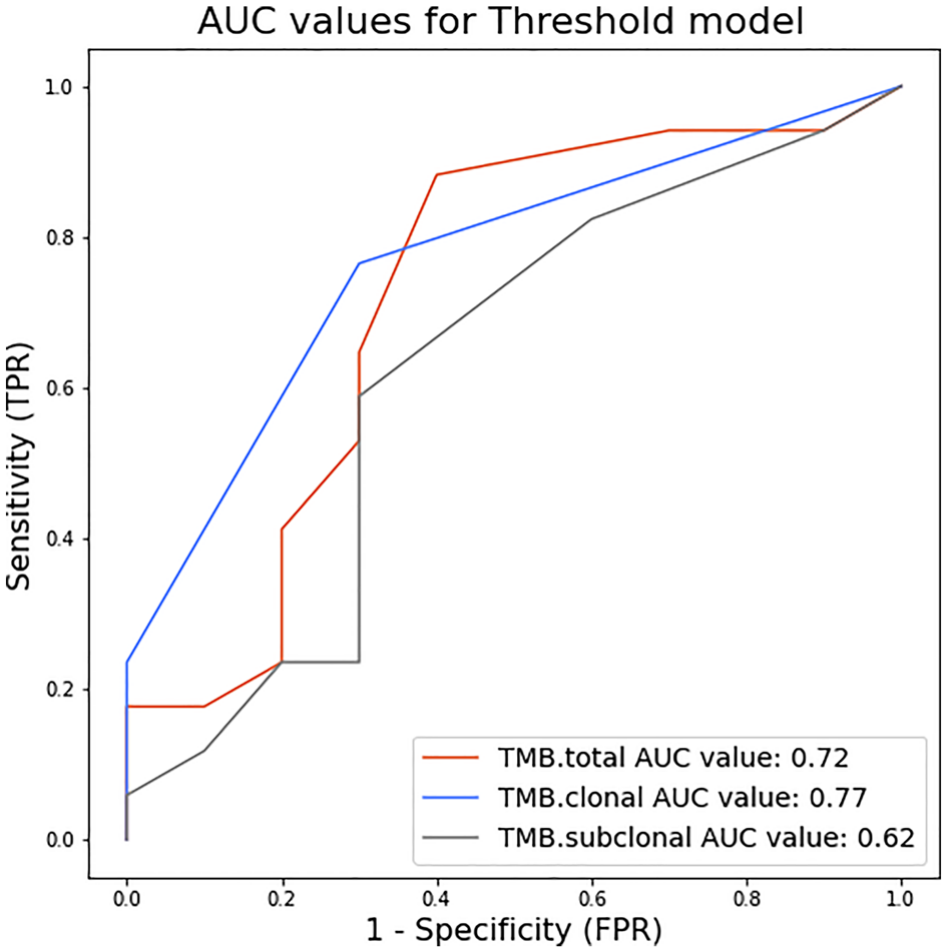
Clonal TMB performs better in separating the two response groups in a threshold model than total TMB. Thresholds to separate response groups in steps of TMB = 1 mut/mB were applied. Sensitivity or true positive rate (TPR) was calculated as the number of true positives divided by the number of true positives + false negatives. Specificity or false positive rate (FPR) was calculated as the number of true negatives divided by the number of true negatives plus false positives.

To better understand the possible implications for the prediction of response to ICI, we applied different TMB thresholds in steps of 1 and we classifed the patients into responders (observed TMB above threshold) and non-responders (observed TMB below threshold). Then, we calculated the true positive and true negative rate for each threshold. Clonal TMB performed best in the threshold model with an AUC of 0.77, followed by total TMB with an AUC of 0.72 and subclonal TMB with an AUC of 0.62 (Fig. 3). Clonal TMB was particularly useful to identify true responders while keeping the false positive rate low (specificity 0.8–0.9).

**Figure 4:**
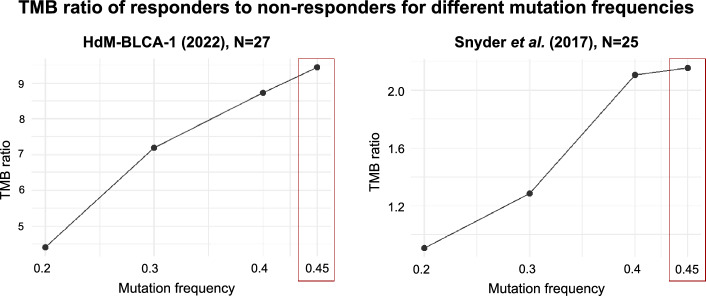
Effect of mutation frequency on the discrimination between responders and non-responders. TMB ratio: ratio of the median TMB of responders versus the median TMB of non-responders. Mutation frequency: minimum variant allele frequency that we consider to calculate TMB. The difference in TMB between responders and non-responders increases with mutation frequency, with maximum values at a mutation frequency of 0.45 in both cohorts.

Inspection of available mutation data from an independent urothelial cancer cohort, published by Snyder et al*.*, also indicated that mutations occurring at high frequencies were especially important in explaining the response^8^. As no information on tumor cell purity or cell fraction was available for this dataset, we used the variant allele frequency (VAF), instead of the cancer cell fraction (CCF), as a proxy of mutation frequency. Similar to what we observed in our cohort, the difference between the median TMB values of responders to non-responders increased with increasing VAF (Fig. 4). The maximum difference was at VAF > 0.45, which essentially represents clonal mutations.

### The positive relationship between predicted neoantigens and response mirrors that observed for TMB

One likely explanation for the association of TMB with response to treatment is that a subset of these mutations will generate neoantigens that trigger an immune system response against the tumor. Among neoantigen presentation, binding affinity is thought to be the most selective step^2^. We predicted the MHC I binding affinity of all possible peptides originating from missense mutations in the tumors using the software NetMHCpan 4.0^31^. Because MHC I receptors are highly variable among individuals, and each molecule has different epitope affinities^32^, we first computed the human leukocyte antigen (HLA) genotype for each patient and then used the specific HLA subtypes for the predictions. Following the NetMHCpan recommendations, we defined the set of putative MHC I bound peptides as those within the 2% top binding rank.

**Figure 5:**
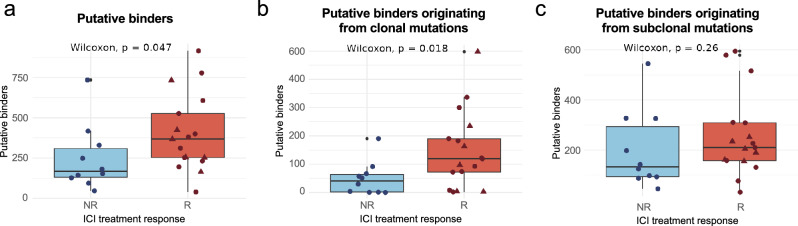
The positive relationship between predicted neoantigens and response mirrors that observed for TMB. (**a**) Relationship between the number of putative binders and the response to the treatment. Responders have a significantly higher number of putative binders than non-responders (Wilcoxon test, p value = 0.047). (**b**) Relationship between putative binders originating from clonal mutations and response to ICI treatment. Number of putative binders originating from clonal mutations is significantly higher in responders than in non-responders (Wilcoxon test, p value = 0.018). (**c**) Relationship between putative binders originating from subclonal mutations and response to ICI treatment. No significant difference can be observed for the number of putative binders originating from subclonal mutations between responders and non-responders. *NR* no responders, *R* responders, triangle shape represents complete responders among the group of responders.

Regarding the relationship with ICI treatment response, we observed significantly more binders in responders than in non-responders (p value = 0.035, Fig. 5a). The difference became more significant when only considering clonal mutations (p value = 0.015, Fig. 5b). In contrast, no significant difference between treatment groups was found in the case of subclonal mutations (p value = 0.13, Fig. 5c). Similar conclusions were drawn when using the concentration that inhibits 50% binding of the fluorescein-labeled reference peptide (IC_50_), or more stringent thresholds (Figure S4), as well as when using a second MHC binding prediction software, MHCflurry 2.0 (Figure S5). The results are consistent with a positive effect of the number of neoantigens that are being presented on the response to the treatment.

Factors other than the peptide MHC binding affinity can also influence neoantigen presentation and immunogenicity^2^. One such factor is the stability of the peptide-MHC (pMHC) complex^33,34^. However, we did not find any significant difference between the number of predicted highly stable pMHC complexes and the response to treatment (Figure S6). Another approach to predict a peptide’s relative immunogenicity is its differential agretopicity index (DAI)^35^. This index measures the difference in the binding affinity of the mutated peptide compared to its non-mutated counterpart. We observed that mutated peptides in non-responders tended to show lower DAI values compared to those in responders but, again, this trend did not achieve statistical significance (Figure S6).

We then asked whether the difference between responders and non-responders could increase in the case of mutations that generated new binders. Using a binding rank < 2% threshold, we identified a total of 2175 new putative peptide binders created by cancer-specific mutations, and another 1901 peptides in which the mutation caused the peptide to lose its ability to bind to MHC I. The new binders were enriched in hydrophobic amino acids, including tyrosine (Y), phenylalanine (F), leucine (L) and tryptophan (W), as well as histidine (H), when compared to mutations causing no change in binder status (Figure S7). In contrast, mutations associated with loss of binding to MHC I were enriched in cysteine (C) and, to a lower extent, glycine (G). We also observed that amino acid replacements that changed the binding status of the peptide tended to be located in the 2nd and 9th position of the peptide. These results are consistent with previous observations that hydrophobic residues are associated with increased immunogenicity^36,37^, and that the 2nd and 9th amino acids of the peptide are anchor positions to the MHC I receptor^37,38^. When responders and non-responders were compared, we again observed increased separation between the groups for clonal mutations compared to other types of mutations (Figure S8). The magnitude of this effect, however, was not larger than when all possible kinds of neoantigens were considered.

We also examined whether, independently of the response, the loss of binder mutations was relatively more frequent among clonal mutations than subclonal ones, this could be expected if this type of mutation is particularly favored in the initial stages of cancer when mechanisms to avoid immune system surveillance might be weaker. We found that, while the ratio of the number of loss of binder mutations versus gain of binder mutations was indeed higher for clonal than for subclonal mutations (0.93 versus 0.84, respectively), the difference was not statistically significant.

### Pathways associated with response and immune response markers

**Figure 6:**
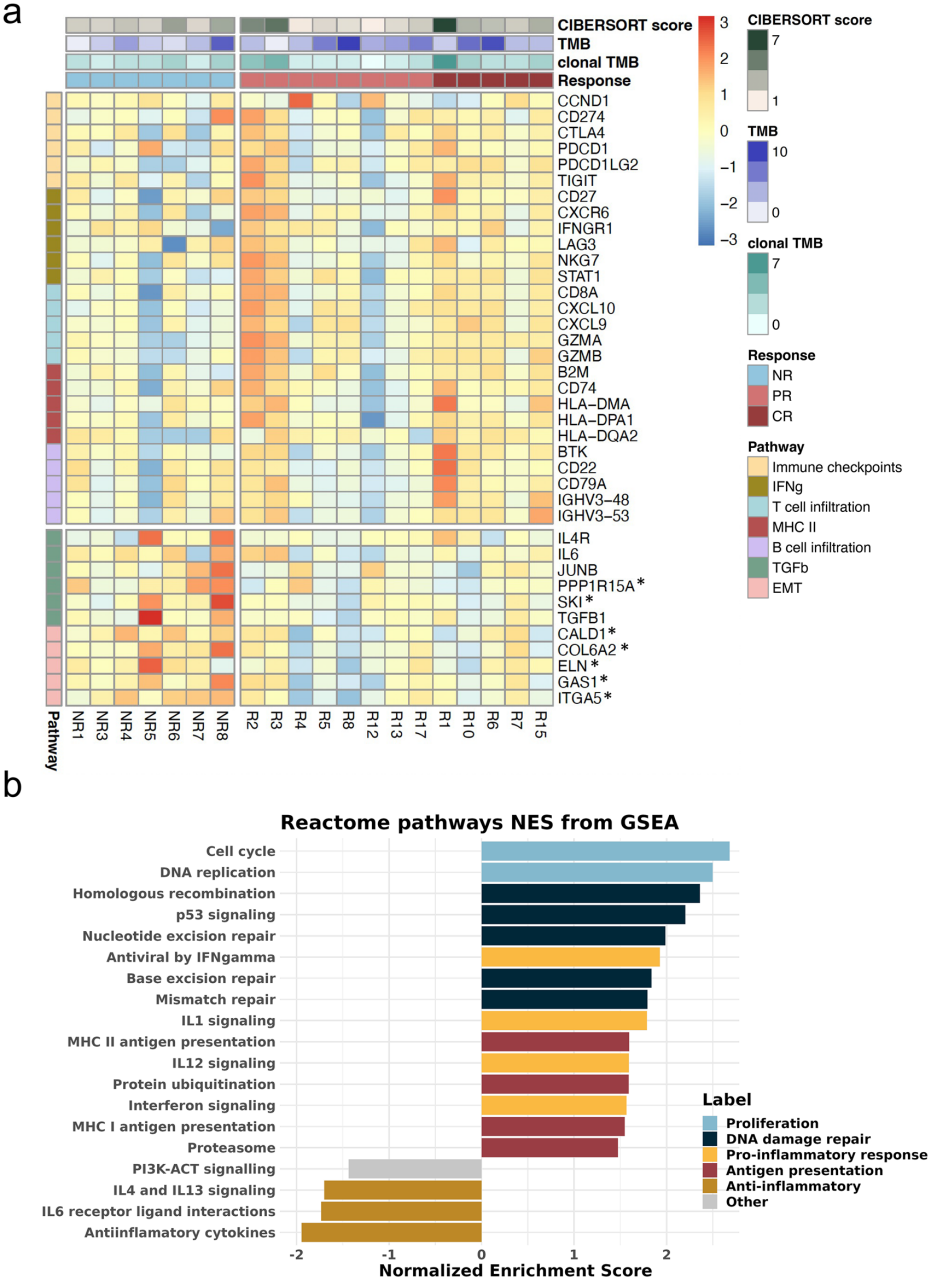
(**a**) Responders with low clonal TMB show expression of genes connected to immune infiltration, while non-responders have higher expression of immune suppression. Gene expression for selected marker genes sorted by pathways. Expression values are normalized and log2cpm transformed. The heatmap is scaled by row. Annotation bars show immune infiltration as CIBERSORT score, tumor mutational burden (TMB) and clonal tumor mutational burden (clonal TMB) and response to ICI treatment. Columns indicate the patient tumor samples. *NR* no responder, *PR* partial responder, *CR* complete responder. Only a few genes were statistically significant in the DE analysis (*p value < 0.05). (**b**) REACTOME pathways connected with proliferation, DNA damage repair, antigen presentation and pro-inflammation are significantly enriched in responders, and anti-inflammation pathways are enriched in non-responders. Normalized enrichment score for selected pathways significantly related to response obtained from GSEA analysis (adjusted *P* < 0.05; comparing 13 responders and 7 non-responders). The complete list of pathways with the included genes is provided in Additional file 3.

In addition to the aforementioned genomic characteristics of neoantigens and the peptide-MHC complex, the tumor environment and other molecular mechanisms are known to play a crucial role in the activation of an immune response. We used the gene expression data to impute tumor-immune cell infiltration abundances using CIBERSORT. We found no significant differences in the overall immune infiltration score between responders and non-responders, but the former group had a significantly higher fraction of CD4 memory-activated T cells (p = 0.029, Figure S9a). Next, we examined the gene expression patterns of different immune markers and gene expression signatures (RNA-Seq data for 20 patients, 13 responders, 7 non-responders). We found that the median expression value of pro-inflammatory markers, immune checkpoints and MHCII antigen presentation genes, tended to be higher in responders than non-responders (Fig. 6a, Figure S9b). In their multivariable predictor model, Litchfield et al.^18^ reported gene expression values of cytokine *CXCL9* to be among the strongest predictors for ICI response. In our cohort, *CXCL9* had more than twice the median value in the responders group (Figure S9b). Other markers related to CD8 T cell immune response, such as *CD45*, *CD8A*, and interferon-gamma pathway genes showed a similar expression pattern. Genes involved in B cell-mediated immunity and MHC class II also tended to be enriched in responders. On the contrary, gene expression of the transcription growth factor beta (TGF-β) was found to be decreased in responders compared to non-responders (Fig. 6a). This result is expected given that TGF- β is generally associated with an immunosuppressive effect^39^.

Analyzing the expression patterns of combinations of genes instead of individual genes can sometimes provide a clearer signal. Using gene set enrichment analysis (GSEA) identified several pathways significantly enriched in the responders group (FDR p value < 0.05). This included DNA damage repair (DDR), proliferation, apoptosis, ubiquitination and pro-inflammatory functions upregulated in responders (Fig. 6b, Figure S10). These pathways are very interrelated and are expected to play a role in ICI response. Additionally, non-responders were characterized by higher expression of anti-inflammatory cytokines like IL4 and IL6, extracellular matrix organization and TGF-β related pathways (Fig. 6b, Figure S10). Analyses performed grouping the genes by Gene Ontology (GO) classes or using molecular pathways produced consistent results (Figures S10, S11a).

We then investigated if there were any differences in gene expression patterns between complete responders (n = 5) and partial responders (n = 8). We found significant differences at the level of T cell-mediated and humoral immunity (Figure S11). Therefore, immune players beyond the classical MHC-I-CD8 T axis, such as those mediated by MHC class II, might be important.

Another interesting observation was that the subset of responders that had the lowest number of mutations (samples R1, R2 and R3; TMB < 3.5 mut/Mb) also had the highest levels of T and/or B cell infiltration markers (Fig. 6b). These patients would most likely have been missed by any TMB threshold method (the majority of the responders had TMB > 5 mut/Mb) but could have been identified using a signature based on immune markers.

## Discussion

Our findings indicate that the number of clonal non-synonymous mutations (clonal TMB) is the strongest predictor of the response to ICI in advanced urothelial cancer, suggesting that focusing on this biomarker could significantly improve the identification of the patients who are more likely to benefit from treatment. The results are well-aligned with previous findings for melanoma and non-small cell lung cancer, in which the burden of clonal neoantigens and a low intra-tumor heterogeneity have been associated with an increased response to ICI^15,16^. In the study of Miao et al., which included a range of cancer types including urothelial cancer, the authors reported that the number of clonal non-synonymous mutations was significantly higher in complete responders than in partial responders or non-responders^17^. Instead, we could not see differences in the number of clonal mutations between complete responders and partial responders. Finally, Litchfield et al. (2021) constructed multivariate predictive models and observed that models considering only TMB and *CXCL9* attained AUC values between 0.63 and 0.79 depending on the test cohort, which was only marginally lower than the full model containing eleven different variables^18^.

We found that the separation between responders and non-responders in our cohort increased as we considered mutations with a higher allele frequency, the same effect being observed using data from an independent urothelial cancer cohort by Snyder et al.^8^. Using a mutation threshold predictive model, the AUC values for clonal TMB were higher than those for total TMB (0.77 versus 0.72). In addition, we found that the ratio of clonal versus subclonal mutations was significantly higher in responders than in non-responders, suggesting that tumors in the responder group are less heterogeneous. A study using a UV-induced mouse melanoma model showed that tumor heterogeneity diminishes the immune response^16^. Taken together, the results indicate that the predictive power of TMB can be significantly increased if we only consider clonal mutations.

The APOBEC signature has been previously associated with the response to ICI in different types of cancer including urothelial cancer^17,38^. APOBEC-mediated mutagenesis becomes activated in early disease stages^21,24,40^ and has been associated with mutation hotspots of several cancer driver genes^41^. In urothelial cancer, the APOBEC signatures are suggested to drive high TMB by introducing genomic instability^42^. Additionally, both the APOBEC signature and high TMB are related to better survival in urothelial cancer patients, independently of treatment and in the context of anti-PD-L1/PD-1 ICI^42,43^. In our study, we did not find a significant positive association between APOBEC and the response to treatment, except when we measured the APOBEC signature specifically in clonal mutations. A potential explanation could be that APOBEC mutations are associated with increased peptide hydrophobicity, as shown by Boichard et al.^38^. This could increase the likelihood of generating immunogenic neoantigens^36,37^.

A detailed inspection of the mutations revealed that many known oncogenes and tumor suppressor genes were frequently mutated in our patient cohort. The topmost mutated genes were *ELF3, KMT2D, ARID1A, TP53* and *PRKDC*; these genes were mutated in 6 patients or more (> 20% of the patients). Interestingly, a recent study found that recurrent somatic mutations in several cancer-related genes and pathways, including the MAPK signaling and TP53 cycle pathways, increased the predictive power of the predictive models over just using TMB. Given that a large portion of the mutations in these genes are clonal, these results also point to the importance of tumor clonality in the response to immunotherapy.

We also examined the relationship between the number of predicted MHC-I binding peptides per patient and ICI treatment response as described in previous studies^31,44^. We found that, similar to TMB, the clonality of the mutations generating the predicted neoantigens increased the difference between response groups. For a subset of the somatic mutations, the mutation increased the binding of the peptide to MHC I above the threshold, potentially generating a new binder. Again, we found that the number of new binders was significantly higher in responders versus non-responder in the case of clonal mutations. Further studies based on experimental data might help disentangle the importance of different types of neoantigens. It has been reported that mutations are more likely to be observed in tumors if the resulting peptides have low affinity for the patient’s MHC I receptors^45^. Although we observed a tendency for increased loss of binding mutations in clonal mutations versus subclonal ones, the results were not conclusive. We expect, however, that these analyses will encourage future studies to understand selective processes involved in immune evasion.

The gene expression analysis of immune markers and immune cell invasion indicated that T cell immune infiltration is an important factor in determining response to the treatment, as found in other studies^8,18,39^. A high neoantigen load will not elicit an immune response if the tumor is not invaded by T cells and consequently, the ICI treatment will be ineffective. Using gene set enrichment we observed that genes involved in proliferation, DNA damage response, antigen presentation, and pro-inflammatory responses were significantly associated with response to treatment. Additionally, we saw higher immune infiltration of CD4 memory T cells as imputed by deconvolution, and increased expression of CD8 T cell markers in responders. High tumor infiltration of CD8 T cells has been associated with improved clinical outcome through the activation by MHC I presented antigens in urothelial bladder cancer^46^. On the contrary, T regulatory (Treg) cells are described to be tumor-promoting agents^47^. Our results indicated a negative association between ICI response and both Treg and Tfh cells, although it did not achieve statistical significance. The latter type of T cells expresses a large number of PD-1 on their surface. It has been suggested that anti-PD-1 treatment could cause immune-related adverse events (ieAEs) by hyperactivation of Tfh, provoking autoimmunity^48^. High levels of Tfh cells have been recently correlated to ieAEs in urothelial bladder cancer patients treated with PD-L1^49^. The role of B cells in ICI therapy in urothelial cancer has been sparsely studied, despite their high expression of PD-L1^50^. While a positive correlation between B cells and improved survival was suggested for different cancer types^51,52^, other studies reported B cell content not to be associated with response to anti-PD-1 in melanoma^53^. Our results indicate that B cell infiltration levels are quite heterogeneous among responders. We further observed that expression of TGF-β tended to be lower in responders than non-responders. The TGF-β signature is associated with fibroblasts in cells excluded from the tumor parenchyma and has been previously associated with a lack of response and resistance to immunotherapy^39^.

Combining information from WES and RNASeq might help to better discriminate between responders and non-responders. In our set of responders, there were three patients with an abnormally low number of missense mutations. Interestingly, these three patients were also the ones with the highest immune infiltrate based on the gene expression analysis, indicating that, at least in these cases, a high number of immune cells was the key. However, the interplay between different variables might be quite intricate. The use of machine learning approaches to build predictive models could be a promising approach, but this requires a large amount of data, and the accuracy of the predictions is still relatively low. In linear regression models, the effects of different variables are additive, which hampers the identification of different classes of responders. Models capable of detecting the biology underneath the data, and the dependencies between different variables, need to be developed.

Finally, a few important limitations should be mentioned. The pipeline focused on missense SNVs, without taking indels or splice-isoforms into account. Peptides occurring from such mutations are expected to be highly immunogenic and they could be contributing in a significant manner in explaining the response to immunotherapy^54^. However, they are much less frequent than missense mutations, and larger cohorts would be necessary to investigate their importance. Regarding the peptide-MHC complex analysis, we only considered MHC class-I-restricted neoantigens as, up to date, the prediction algorithms for MHC II are less reliable and with lower accuracy^2^, but MHC-II-mediated immune response might also be relevant in ICI response^55,56^. It is worth mentioning that antigen preprocessing steps, such as peptide cleavage, its affinity to TAP protein, or ERAP trimming, are also important to predict neoantigens. To account for peptide processing, we also performed searches with MHCflurry 2.0, reaching similar conclusions as those obtained with NetMHCpan 4.0^2,31,57^. Finally, the bulk nature of our data (both WES and RNASeq) restricts all of our results to in-silico estimations. Future studies using single cell profiling will be needed to confirm our findings related to clonality and cell-type abundances. All neoantigen properties were based on predictions, which poses important limitations to the interpretation of the results. While performing immune-peptidomics experiments would provide a more realistic view of the neoantigen landscape, these experiments are not possible with FFPE samples.

This study presents a comprehensive and systematic analysis of a set of different biomarkers and their role as predictive biomarkers of response to ICI treatment in advanced urothelial cancer patients. We provide evidence that clonal TMB is a stronger predictor of response to ICI than total TMB. The results also suggest that, in some cases, patients with low TMB can respond to the treatment; this is generally associated with high levels of immune markers. Our study provides new data supporting that more homogeneous cancers in terms of TMB might be more likely to respond to ICI, and suggest that non-additive effects of different variables should be considered in future efforts to develop predictive models. Building predictive models able to combine different possible responder profiles might help address some of the present challenges for the management of advanced urothelial cancer with ICI.

## Material and methods

### Patient data

The data analyzed here was derived from biological samples of 27 metastatic urothelial cancer patients (4 female and 23 male patients) treated in Hospital del Mar with anti-PD-1/PD-L1 ICIs. This study was approved by the Institutional Review Board of Hospital del Mar. For 27 patients, whole exome data was obtained from tumors and blood samples Patients were classified as having clinical benefit (R—responders) if they had a partial or complete response in tumor burden, or having no clinical benefit (NR—non-responders) if they had progressive disease as the best response to treatment. Radiologic responses or progressive disease were defined as per the Response Evaluation Criteria In Solid Tumors (RECIST) criteria 1.1. Of the 27 patients, 17 were responders to the ICI treatment with 5 being complete responders. DNA and RNA extraction from formalin-fixed paraffin-embedded (FFPE) tumor specimens and blood samples was performed according to our experience^58,59^. Whole exome sequencing (WES) and RNA-sequencing (RNASeq) were done by the Centro Nacional de Análisis Genómico, Barcelona (CNAG). Sample coverage was analyzed by qualimap (Version 2.2.1) with default parameters and copy number analysis was conducted using ControlFreeC (Version 5.6) using the BAF options.

### Variant calling

The preprocessing of the raw sequencing data was conducted at CNAG following the best practice GATK4 pipeline (version 4.0.8). Sequence reads were mapped to the reference genome (hs37d5) using BWA (version 0.7.17) to obtain SAM/BAM files sorted by coordinates. To mitigate biases introduced by data generation steps such as PCR amplification, duplicates were marked, and base quality scores were re-calibrated, as variant calling algorithms rely heavily on the quality scores assigned to the individual base calls in each sequencing read. Mutect2 (version 40.1.2) and Strelka2 (version 2.9.10) were used as variant callers comparing somatic and germline samples for each patient. Mutations were annotated using VEP (Version 104). SNVs that pass the default filters of both, Mutect2 and Strelka2, were further filtered for population-wide allele frequency under 5% (gnomAD), a minimum sample depth of 30, and a minimum alternative allele depth of 3. Further analyses were performed focusing on missense mutations to facilitate the comparison of mutated and non-mutated peptides. TMB was measured using the function tmb() of the maftools R-package (version 2.10.0)^22^, estimating the number of non-synonymous mutations per capture size of 50 megabases as this is the target region of the kit used. The TMB threshold model was built by applying different thresholds in steps of 1 to the dataset, separating the patients into responders (TMB above threshold) and non-responders (TMB below threshold). From there, the number of correctly and misclassified patients was used to obtain specificity (True positive rate = true positives/(true positives + false negatives) and sensitivity (1 − True negative rate = 1 − (true negatives/(true negatives + false positives)) for each threshold.

### APOBEC enrichment estimation

To assess the apolipoprotein B mRNA editing catalytic polypeptide-like (APOBEC) mutational signature enrichment, the trinucleotideMatrix function of the maftools R-package was used^22^. The function compares the enrichment of C>T mutations occurring in TCW motives over the total of C>T mutations in the given sample to a background of occurring cytosines and TCW motives. Samples with an enrichment score > 2 and p value < 0.05 were considered significant.

### Clonality

Clonality was defined as mutations with a cancer cell fraction (CCF) above 0.9 and it is calculated as:

$${\text{CCF}}={\text{VAF/p}} * (2 * (1-{\text{p}})+{\text{c}} * {\text{p}})$$

c = copy number, p = purity.

Sample-specific tumor purity and copy number are included to adjust for the variable tumor content and copy number changes following Tarabichi et al*.*^60^. Clonal mutations were therefore defined as SNVs with a *CCF* > 0.9. Tumor purity and local allele-specific copy number were computed using ASCAT (version 3.0). We used default parameters to run ASCAT, except for gamma = 1 as recommended by the developers for WES data^61^. The input for ASCAT was generated using alleleCount (version 4.3.0). The loci file to obtain allele-specific copy numbers was downloaded from the nf-core/sarek pipeline (release 3.1.1)^62^. We excluded all loci not covered by the exome target regions from the loci file by building the intersect of loci and BED file that was used for the WES data.

### Neoantigen and HLA prediction

For each patient, the according 4-digits HLA genotype was determined following the nf-core/hlatyping pipeline (release 1.2.0)^63^ using the HLA genotyping algorithm OptiType (version 1.3.5)^32^. We used blood samples to type each patient's HLA alleles running OptiType at default parameters. Secondly, we computed all possible 9-mer peptides that encompass a mutation, as well as their non-mutated counterpart. This was done using an in-house python script with a sliding window. The peptide sequences were downloaded from Ensembl GRCh37, release 75.

### Binding affinity

To predict the binding affinity of tumor and germline peptides to MHC-I molecules, NetMHCpan (version 4.0) and MHCflurry (version 2.0.4) were used^31,57,64^. By default, NetMHCpan 4.0 labels peptides with a binding rank under 2% as weak binder (WB) and under 0.5% as a strong binder (SB), as defined by the program. The rest of the peptides were predicted to have no binders. We additionally applied a threshold using the predicted concentration that inhibits 50% binding of the fluorescein-labelled reference peptide (IC_50_). Peptides with a binding affinity IC_50_ < 500 nM were labelled to be weak binding and peptides with IC_50_ < 50 nM being strong binding.

By comparing the classification of the mutated peptides and their non-mutated counterparts, we identified cases in which the mutation was predicted to cause a peptide to become a new binder and cases in which the opposite happened (no binder to WB, and WB to no binder, respectively). We compared the frequency of all possible amino acid replacements in these peptides to those occurring in peptides for which no change in binding status was predicted (WB to WB, or no binder to binder). The latter provided an expectation against which to compare the observations. This allowed to identify which amino acids were most strongly associated with the gain of new binders or the loss of existing binders. We performed similar analyses in relation to the peptide position in which the change was observed.

### Binding stability

Binding stability was predicted using netMHCstabpan (version 1.0)^34^. The applied threshold for long binders was 1.4 h following the approach of Wells et al*.*^65^.

### Agretopicity

To rank the mutated peptides by improved MHC-I binding affinity compared to their wildtype counterparts, we calculated the differential agretopicity index (DAI) as described by Duan et al*.*^35^ and used in previous studies^18,66^. Following the approach of Rech et al*.*^44^, a threshold was applied at DAI > 9 for neoantigens with high differential binding affinity compared to their non-mutated counterparts. The threshold for peptides with high DAI was set to 9 as this included only mutated peptides with a binding affinity of more than > 50 nM.

### Gene expression analysis

For 22 of the 27 patients, RNASeq data was generated. Raw sequencing reads were mapped with STAR (version 2.6.0)^67^. Gencode (release 29) based on the GRCh38.p13 reference genome and the corresponding gene transfer format (GTF) file was used. The table of counts was obtained with FeatureCounts function in the package subread (version 1.6.4)^68^ with the previously mentioned GTF file. Genes having less than 10 counts in at least 7 samples were excluded from the analysis. Raw library size differences between samples were treated with the weighted “trimmed mean method” TMM^69^ implemented in the edgeR package (version 3.36.0)^70^. The normalized log2CPMs were used in order to make hierarchical clustering and PCA to assess batch effects and outliers. One sample (R16) was removed from further analysis (Figure S12). For the differential expression (DE) analysis, we used the DGElist object with TMM normalization factors as input for the voom approach of the limma package (version 3.50.0), which models the mean–variance relationship of the log-counts with precision weights. The results of the DE analysis are presented as volcano plots in the supplementary figure S13. We assessed the incursion of surrogate variables and covariates but deemed it unnecessary since the results did not improve. Pre-Ranked Gene Set Enrichment Analysis (GSEA)^71^ implemented in clusterProfiler^72^ package (version 3.18.0) was used in order to retrieve enriched functional pathways. The ranked list of genes was generated using the -log(p.val)*signFC for each gene from the statistics obtained in the DE analysis with limma. Functional annotation was obtained based on the enrichment of gene sets belonging to gene set collections in Molecular Signatures Database (MSigDB, version 7.5). The complete results are provided in Additional data file 3. The association of previously described gene signatures with response was tested using the normalized log2CPM with the GSVA package^73^. TPM values were used for the deconvolution with CIBERSORTx^74^ method with the LM22 gene signature and using B-mode batch correction and absolute mode. The CIBERSORT estimated abundances and the gene signatures scores computed with GSVA are detailed in Additional File 2 and 3, respectively.

### Application of clonality threshold to an independent dataset

As an independent dataset, we analyzed the publicly available mutations of 26 urothelial cancer patients treated with atezolizumab (an anti-PD-L1 antibody) previously published by Snyder et al.^8^. The downloaded somatic mutations were reannotated using VEP (Version 104). Due to the low number of mutations called by Strelka2 (average of 25 mutations/patient, range 11–62) it was not possible to build the intersect of the two callers, Strelka2 and Mutect2, as it was done for the dataset of Hospital del Mar. Instead, the union was built of both lists, following the method described by Snyder et al*.* Mutations were then filtered as described above (gnomAD < 5%, sample depth > 30X, alternative allele depth > 3X). Following Snyder et al*.*, patient 4072 was excluded due to low coverage. Clinical treatment response was evaluated as described above. No information on tumor sample purity and cell fraction were available. Thus, the effect of clonality was estimated by applying different thresholds of minimum tumor variant allele frequency (VAF) from 0.1 to 0.7. Above 0.7, only one responding patient was found to have mutations passing the threshold. The TMB ratio was calculated as TMB in responders divided by TMB in non-responders. To compare these results, the same calculation was repeated for the Hospital del Mar dataset.

### Statistical tests and graphs

All plots included in this manuscript were generated using R (version 4.1.2) and RStudio (version 1.4.1106). For data integration we also used scripts written in python (version 3.5.2). Wilcoxon signed-rank test was used to obtain the significant differences between treatment response groups.

### Ethical approval

The presented study was approved by the institutional Ethics Committee for Clinical Investigation of the Hospital del Mar-IMIM, Barcelona, Spain (2016/6767/l) and conducted in accordance with the principles set out in the World Medical Association guidelines (Seventh revision of the 249 Declaration of Helsinki, Fortaleza, Brazil, 2013) for human subjects involved in medical investigations. All patients signed informed consent for the analysis of tumor biopsies for research purposes and biomarker assessment.

### Supplementary Information

Supplementary Information 1.

Supplementary Information 2.

Supplementary Information 3.

## Data Availability

The dataset generated and analyzed during the current study is available in the EGA repository under the study ID EGAS00001007086.

## Abbreviations

AUC: Area under the curve
APOBEC: Apolipoprotein B mRNA editing catalytic polypeptide-like
BLCA: Bladder cancer
CCF: Cancer cell fraction
CNAG: Centro Nacional de Análisis Genómico, Barcelona
DAI: Differential agretopicity index
DE: Differential expression
DDR: DNA damage repair
FFPE: Formalin-fixed paraffin-embedded
GSEA: Gene set enrichment analysis
GTF: Gene transfer format
HLA: Human leukocyte antigen
ICI: Immune checkpoint inhibitor
MHC: Major histocompatibility complex
MSigDB: Molecular Signatures Database
NK: Natural killer
PD-1: Programmed death-1
PD-L1: Programmed death ligand-1
RECIST: Response Evaluation Criteria In Solid Tumors
RNA: Sequencing (RNASeq)
SB: Strong binder
TCGA: The cancer genome atlas
TGF-β: Transcription growth factor beta
TMB: Tumor mutational burden
VAF: Variant allele frequency
WES: Whole exome sequencing
