# Supplementary material for: The impact of mutational clonality in predicting the response to immune checkpoint inhibitors in advanced urothelial cancer
Source: Sci Rep. 2023 Sep 15;13:15287. doi: 10.1038/s41598-023-42495-2 (PMC10504302; doi:10.1038/s41598-023-42495-2)
Supplement: Supplementary file 1 — Supplementary Information 1. [file 41598_2023_42495_MOESM1_ESM.pdf]

The impact of mutational clonality in predicting the response to immune checkpoint inhibitors in advanced urothelial cancer

Lilian Marie Boll[1*], Júlia Perera-Bel[1*], Alejo Rodriguez-Vida[1,2,3], Oriol Arpí[1], Ana Rovira[1,2,3], Núria Juanpere Rodero[1], Sergio Vázquez Montes de Oca[1], Silvia Hernández-Llodrà[4], Josep Lloreta[1,4], M. Mar Albà[1,5,#], Joaquim Bellmunt[1,6,#]

[1]PSMAR-IMIM Research Institute, Barcelona, Spain.

[2]Medical Oncology Department, Hospital del Mar, Barcelona, Spain.

[3]Centro de Investigación Biomédica en Red de Oncología (CIBERONC-ISCIII), Barcelona, Spain.

[4]Department of Medicine and Life Science, Universitat Pompeu Fabra (UPF), Barcelona, Spain.

[5]Catalan Institute for Research and Advanced Studies (ICREA).

[6]Dana Farber Cancer Institute & IMIM Research Lab., Harvard Medical School Boston Massachusetts.

*co-first authors

#co-corresponding authors

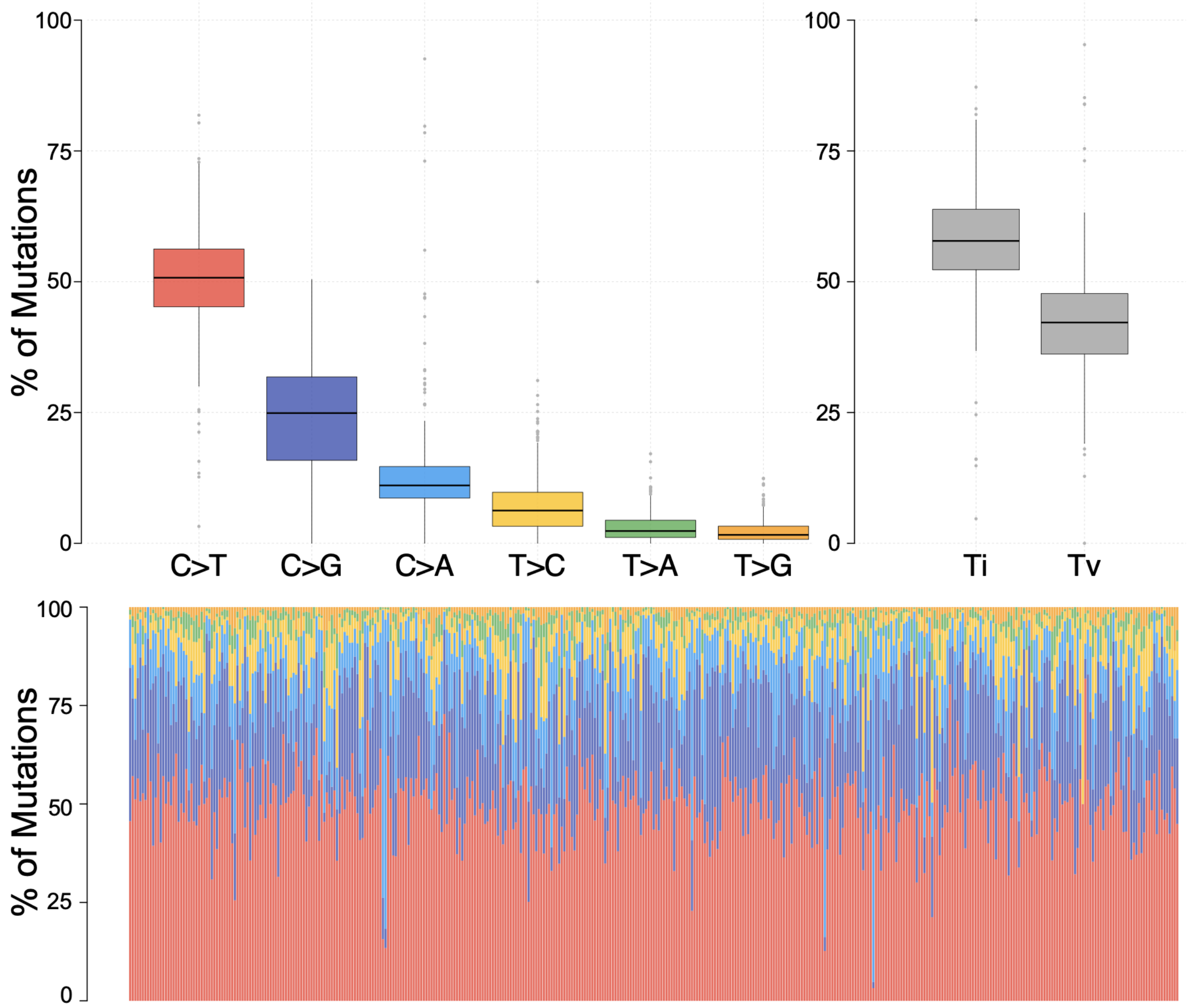

Figure S1. Pairwise nucleotide substitution patterns in the urothelial cancer cohort of the Cancer Genome Atlas (TCGA). The frequency of transitions (Ti) and transversions (Tv) is also shown. The number of samples is 411.

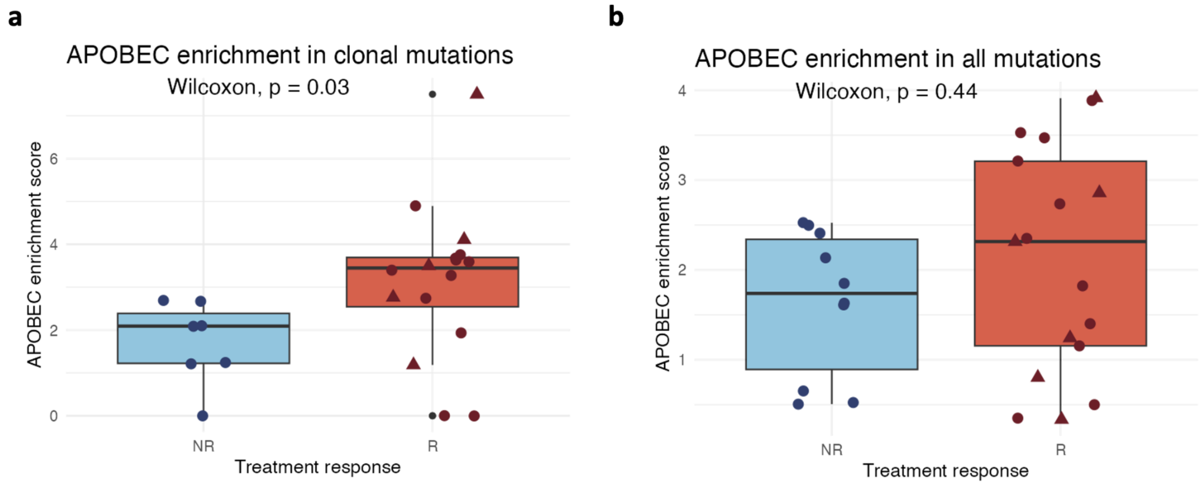

Figure S2. Relationship between APOBEC mutations and response to treatment. a. APOBEC enrichment in clonal mutations. There is a significant positive relationship between response to treatment and the APOBEC enrichment in clonal mutations (p-value = 0.03, Wilcoxon rank sum test). **b. APOBEC enrichment in all mutations.** The differences between responders and non-responders are not statistically significant. NR: no responders, R: responders, triangle shape represents the complete responders among the responder group.

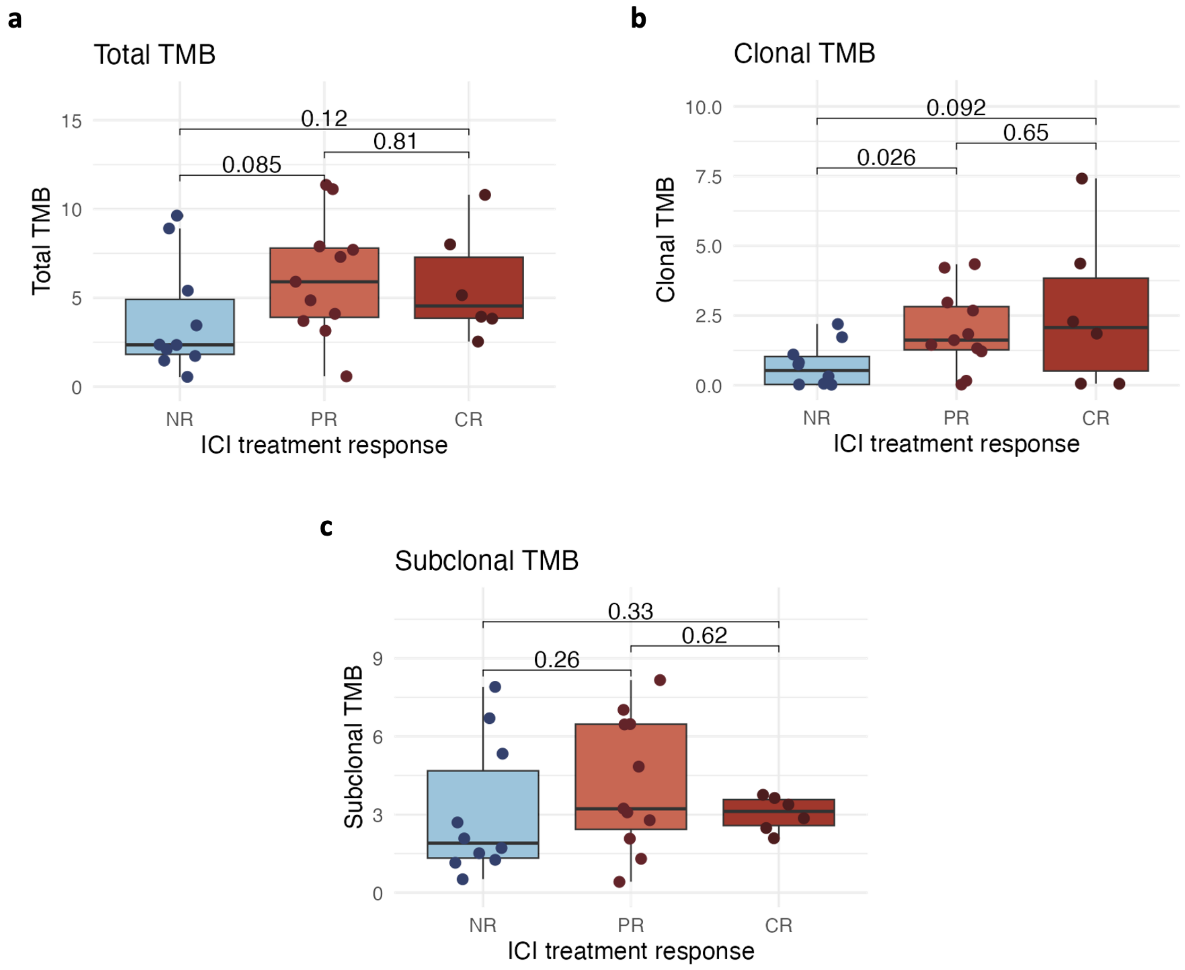

Figure S3. Relationship between TMB and the three different response groups to ICI therapy. a. Relationship between TMB and response to ICI treatment. The differences in TMB values between the three response groups were not significant. **b. Relationship between clonalTMB and response to ICI treatment.** The clonal TMB for partial responders is significantly higher compared to non-responders (p-value = 0.026). The differences in clonal TMB between non-responders and complete responders and partial responders compared to complete responders did not reach statistical significance. **c. Relationship between subclonal TMB and response to ICI treatment.** The differences in subclonal TMB values between the three response groups were not significant. NR: no responders, PR: partial responders, CR: complete responders.

NetMHCpan 4.0

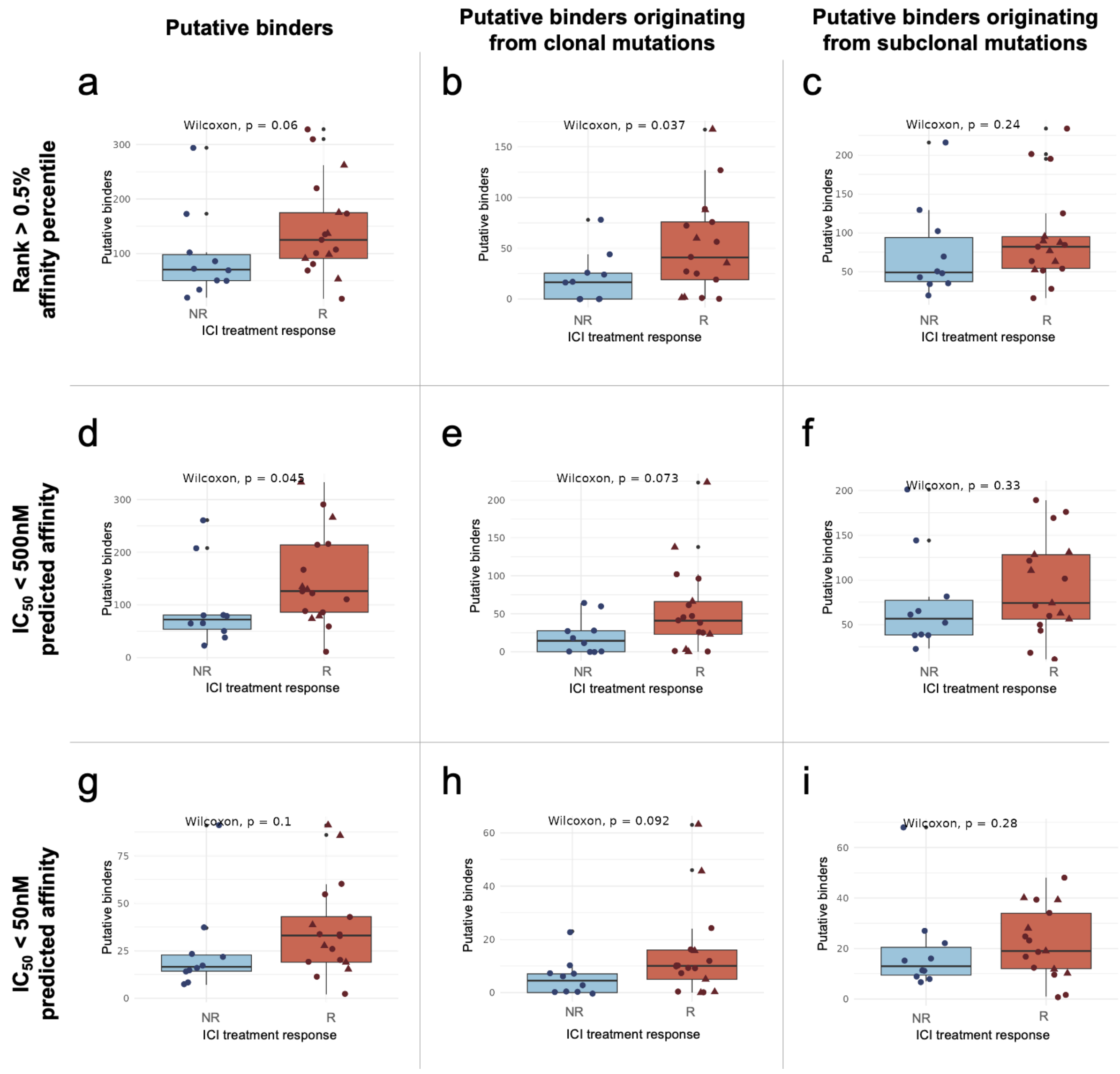

Figure S4. Relationship between ICI treatment response and the number of putative binders predicted with NetMHCpan 4.0. The number of predicted binders tends to be higher in responders than in non-responders. **a-c:** Number of putative binders with predicted affinity rank < 0.5%. **d-f:** Number of putative binders with predicted affinity IC_{50} < 500nM. **g-i:** Number of putative binders with predicted affinity IC_{50} < 50nM. P-values were calculated using the Wilcoxon rank sum test. NR: no responders, R: responders, triangle shape represents the complete responders among the responder group.

MHCflurry 2.0

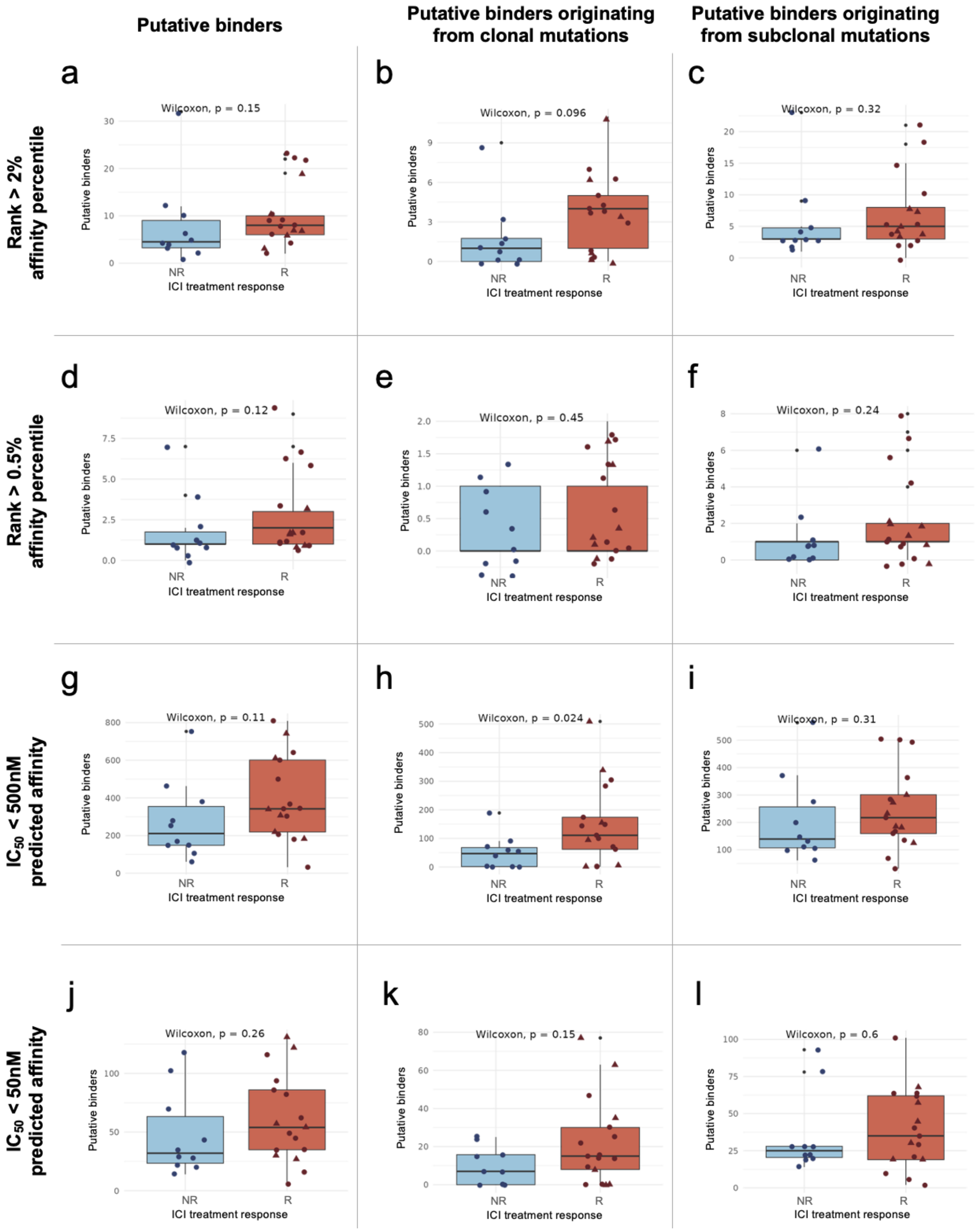

Figure S5. Relationship between ICI treatment response and the number of putative binders predicted with MHCflurry 2.0. The number of putative binders tends to be higher in responders than non-responders. **a-c:** Number of putative binders with predicted affinity rank < 2%. **d-f:** Number of putative binders with predicted affinity rank < 0.5%. **g-i:** Number of putative binders with predicted affinity IC_{50} < 500nM. **j-l:** Number of putative binders with predicted affinity IC_{50} < 50nM. P-values were

calculated using the Wilcoxon rank sum test. NR: no responders, R: responders, triangle shape represents the complete responders among the responder group.

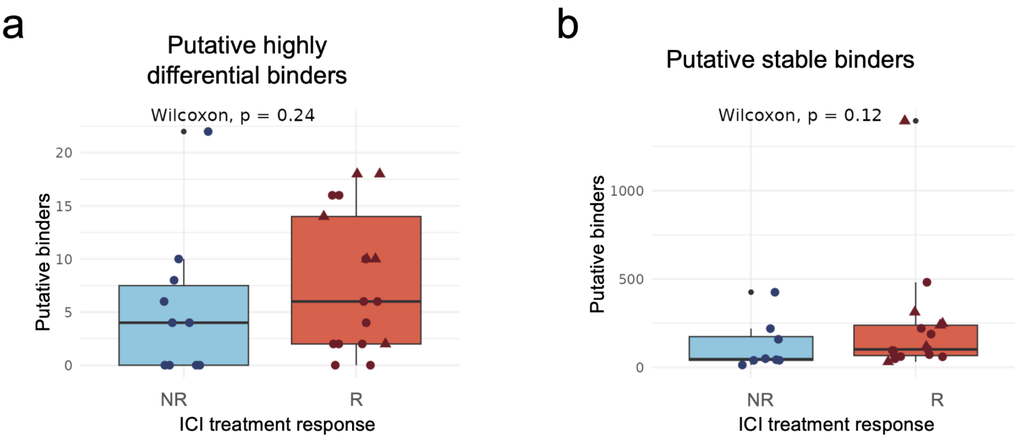

Figure S6. a. Relationship of differential agretopicity index (DAI) and treatment response. No significant difference in highly differential peptides (DAI>9) was seen between response groups. **b. Relationship between the number of predicted stable binders and treatment response.** No significant difference in the number of putative stable binders (binding stability<1.4h) was seen between response groups. P-values were calculated using Wilcoxon rank sum test. NR: no responders, R: responders, triangle shape represents the complete responders among the responder group.

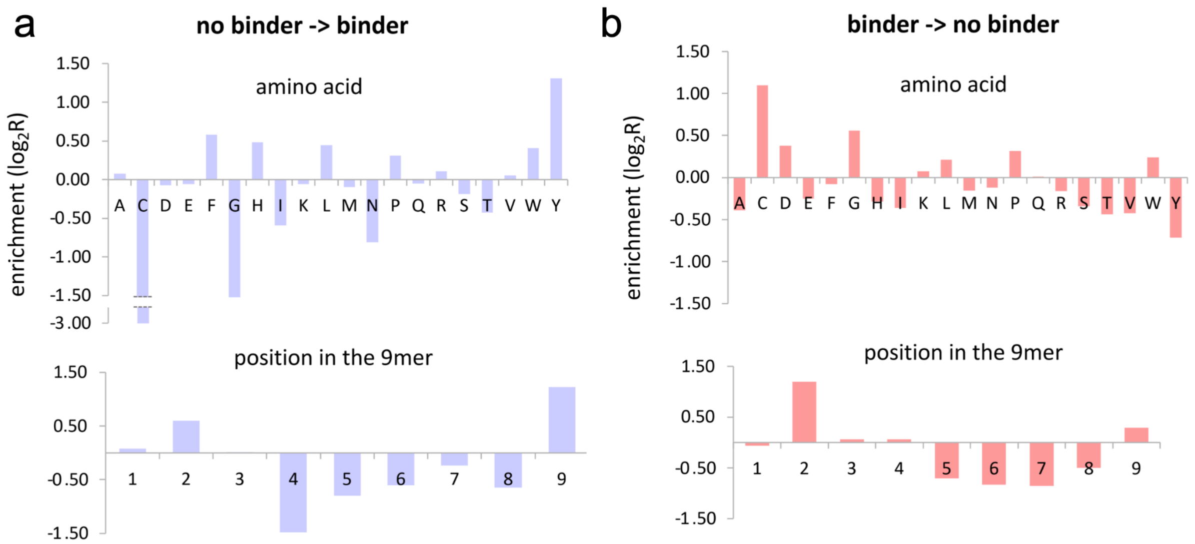

Figure S7. Non-synonymous mutations affect the binding affinity of the peptide. a. Formation of new MHC I binders. Enrichment for different amino acid substitutions (above) or positions in the peptide (below) are measured as the log2 ratio of the substitution frequency in the set of new binders versus the frequency in the set of peptides that do not change binding status. Enriched amino acids using a chi-square test: tyrosine (Y), phenylalanine (F), leucine (L) and histidine (H) at p-value < 10^{-5}, tryptophan (W) at p-value = 0.002872. **b. Loss of MHC I binding capacity**. Enrichment for different amino acid substitutions (above) or positions in the peptide (below) are measured as the log2 ratio of the substitution frequency in the set of peptides associated with loss of MHC I binding *versus* the frequency in the set of peptides that do not change their binding status. Enriched amino acids using a chi-square test: cysteine (C) (p-value < 10^{-5}), glycine (G) (p-value =6.55×10^{-5}).

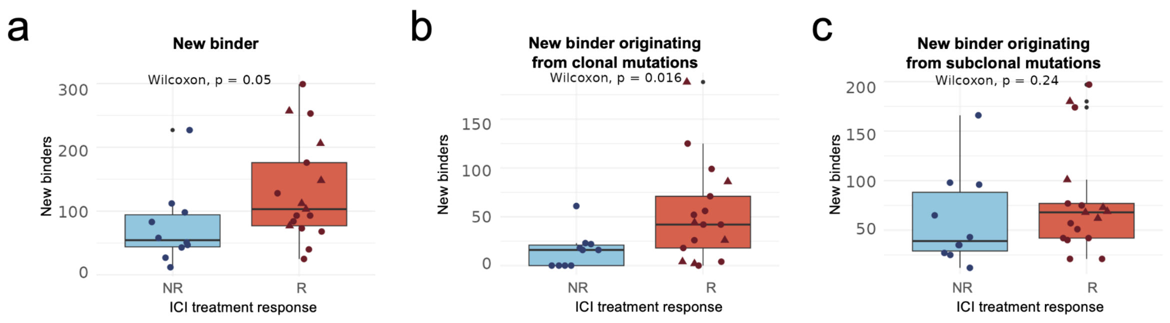

Figure S8. Relationship of number of predicted new binders and treatment response. a. Binders derived from total mutations. Responders have a higher number of new putative binders than non-responders (Wilcoxon test, p-value = 0.05). **b. Binders derived from clonal mutations.** Number of putative binders originating from clonal mutations is significantly higher in responders than non-responders (Wilcoxon test, p-value = 0.016). **c. Binders derived from subclonal mutations.** No significant difference can be observed for the number of putative binders originating from subclonal mutations between responders and non-responders. NR: no responders, R: responders, triangle shape represents the complete responders among the responder group.

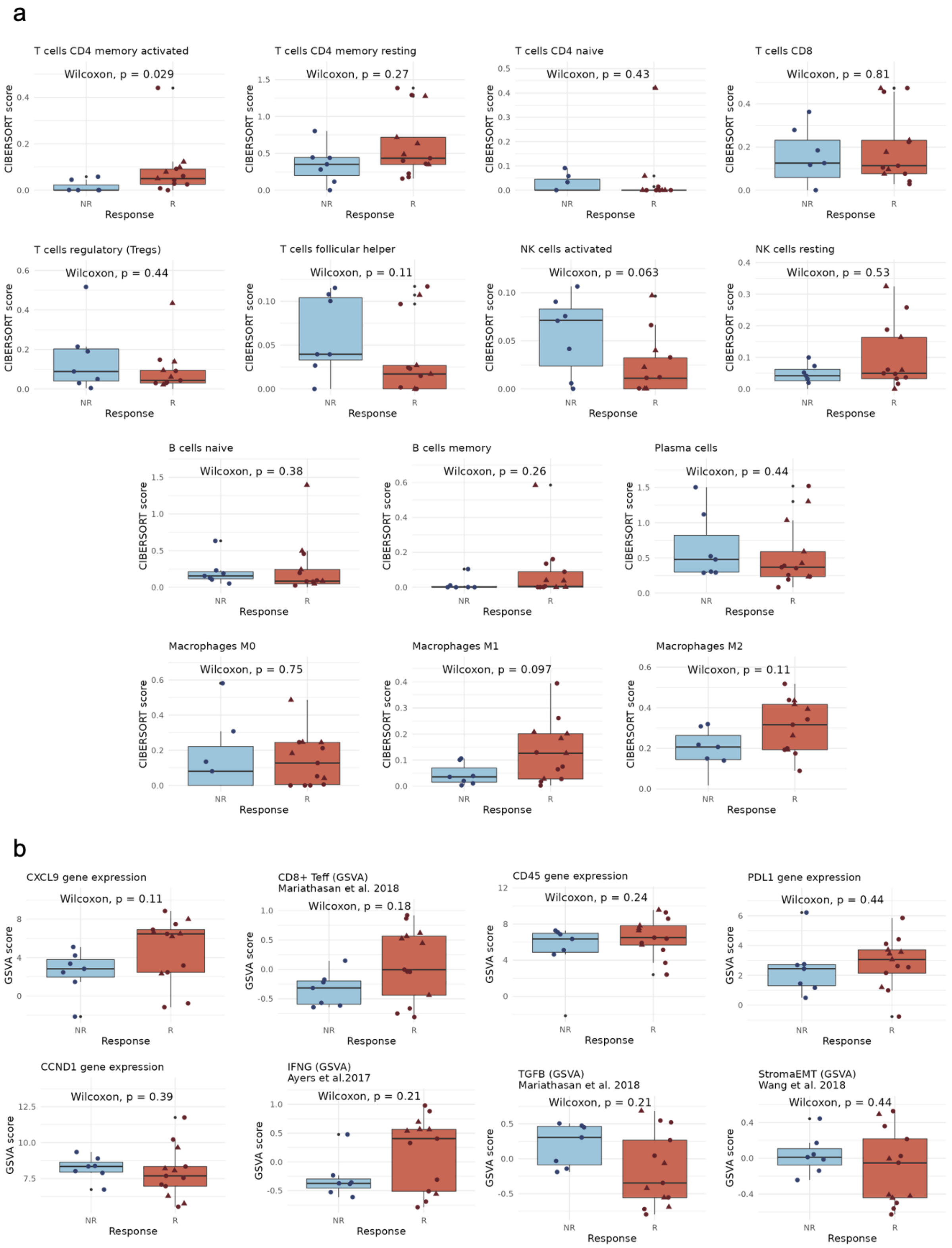

Figure S9. Relation between tumor-infiltrating estimated immune cells abundances and immune biomarkers and treatment response. Boxplots comparing **a. abundance** of immune cells and treatment response and **b.** expression of different immune-related genes (log2CPM) and signatures (GSVA scores). Immune cell infiltration was estimated with CIBERSORT using gene

expression profiles. P-values were calculated using Wilcoxon rank sum test. Details on immune biomarkers and immune cell profiles can be found in the supplementary data file 2. NR: no responders, R: responders triangle shape represents the complete responders among the responder group.

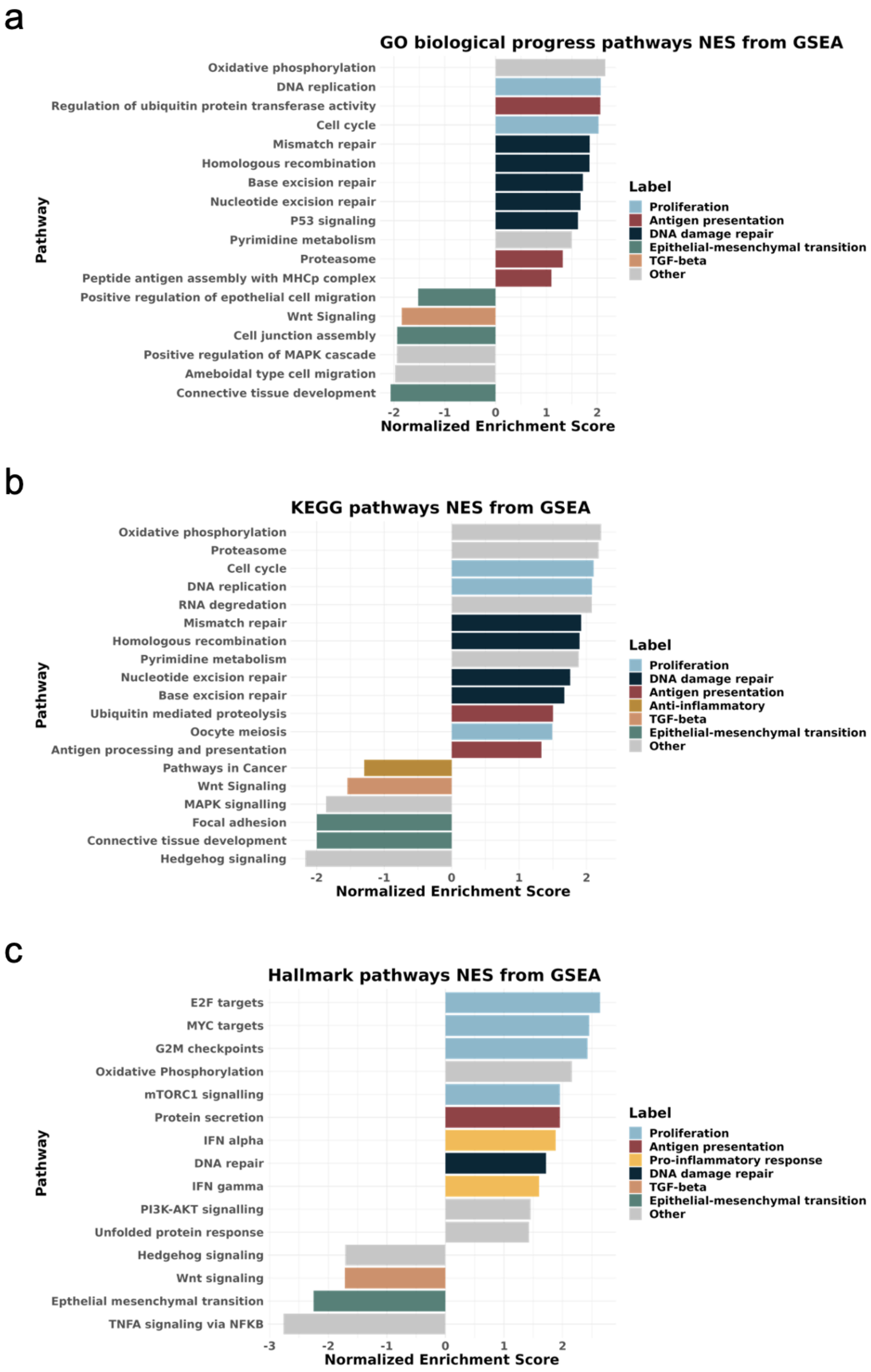

Figure S10. Pathways of gene set enrichment analysis significantly related to ICI response (adjusted $P < 0.05$, comparing 13 responders and 7 non-responders). Selected pathways are shown. The complete list of pathways with their adjusted p-value, normalized enrichment score and the included genes is provided in the Additional data file 3.

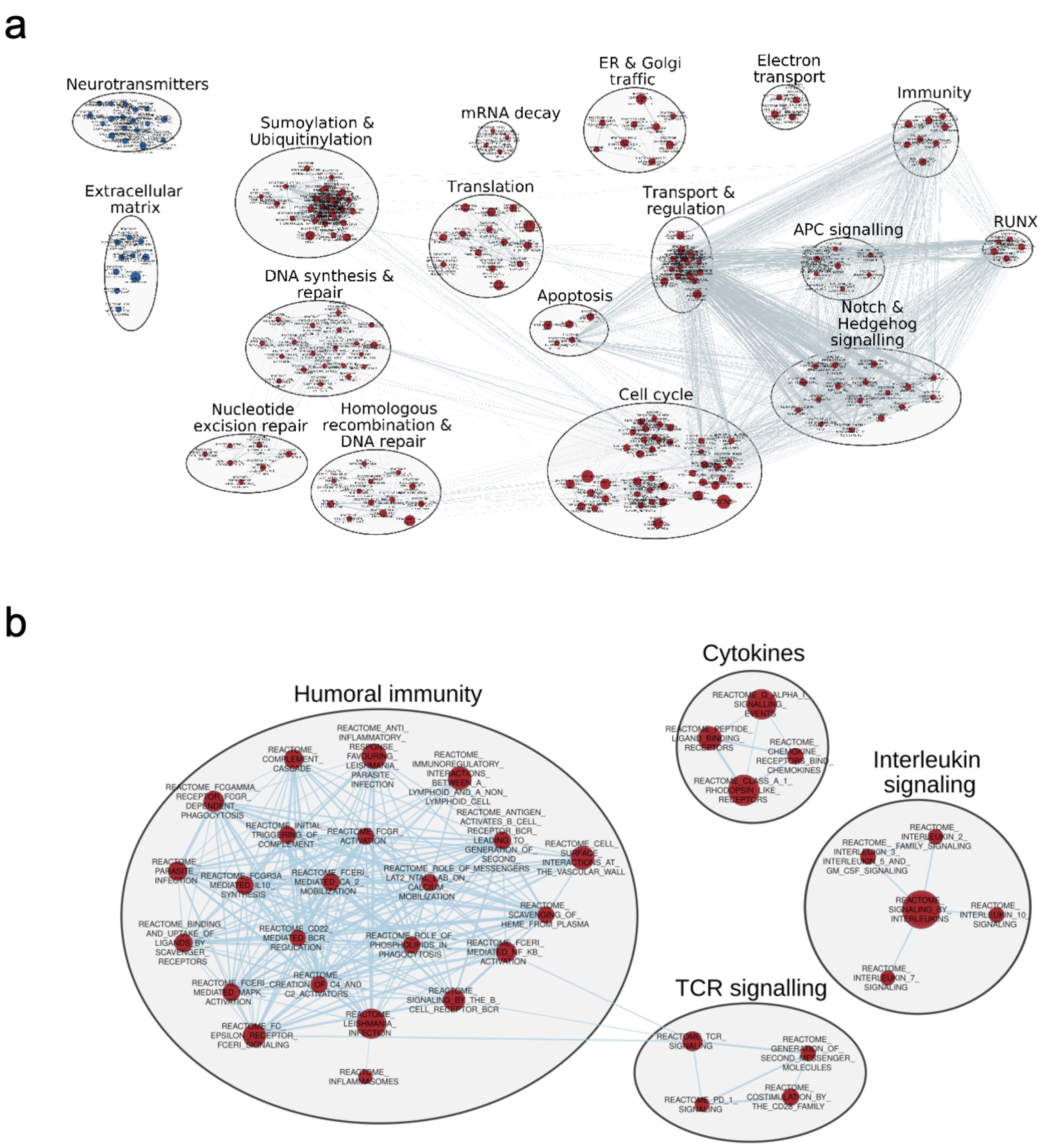

Figure S11. Network of significantly enriched pathways. Enrichment map of GSEA results (adjusted p-value < 0.05) comparing **a.** responders *versus* non-responders and **b.** complete responders *versus* partial-responders. Nodes represent genesets and edges represent the connectivity between genesets (combined metric Jaccard+Overlap >0.375). Red and blue represent positive and negative enrichment scores, respectively.

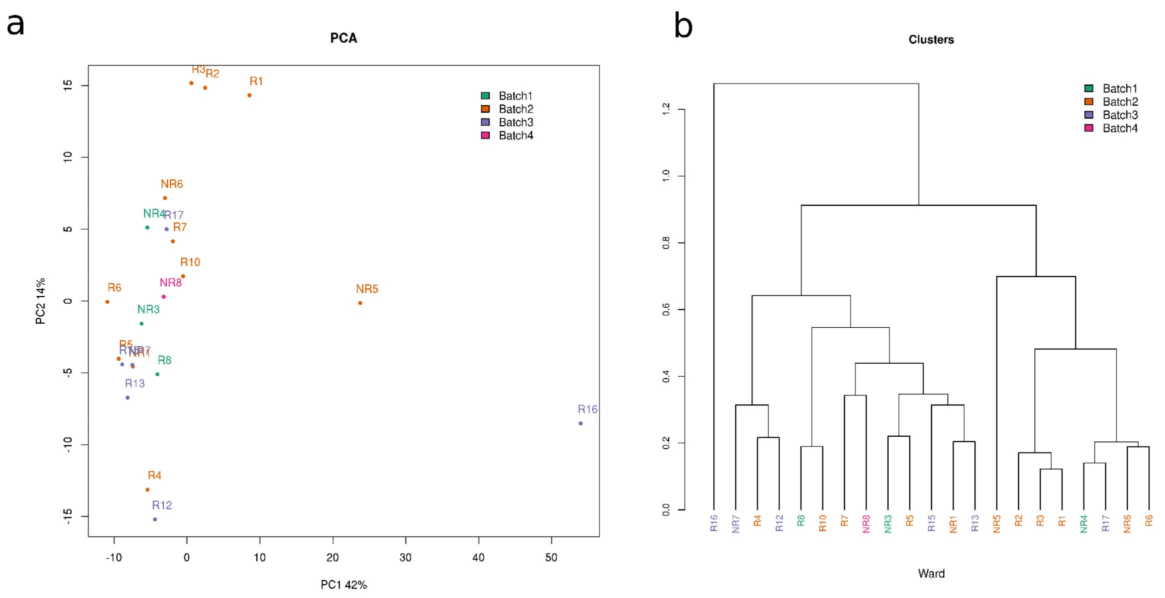

Figure S12. Quality control of RNASeq data. a. PCA using the top 500 most variable genes. **b.** Dendrogram using the top 500 most variable genes, 1-correlation distance and Ward2 linkage method. R16 was detected as an outlier. No batch effect was present.

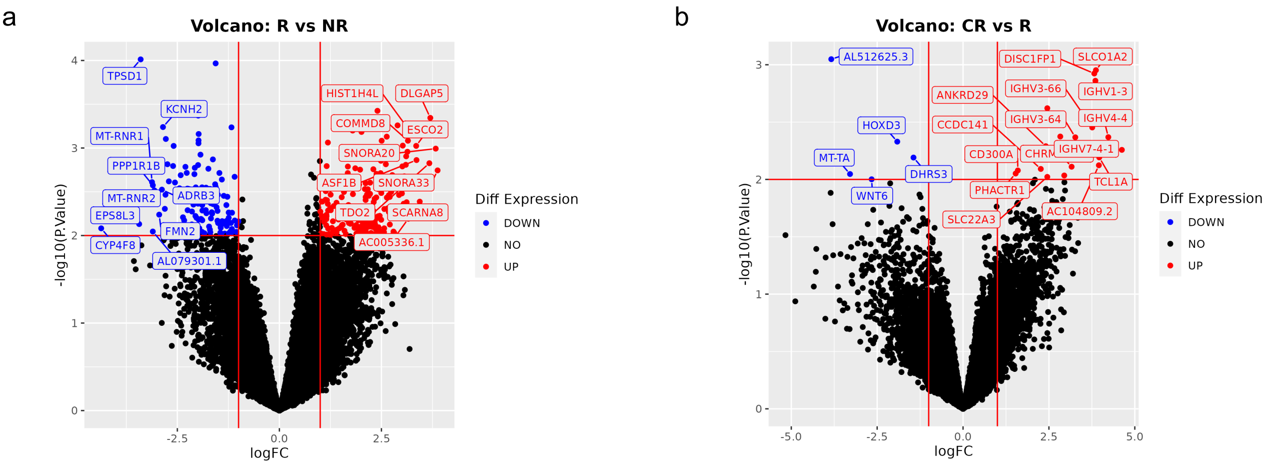

Figure S13. Differentially expressed genes. Volcano plots showing the results of the contrasts: **a.** responders vs non-responders. **b.** complete responders vs partial responders. The thresholds used in the plots are P.Value < 0.01 and |logFC|>1
